# Long Noncoding RNA TOB1‐AS1 Represses Cervical Cancer Cell Proliferation, Invasion, and Migration via the MicroRNA‐27a‐3p/Thioredoxin‐Interacting Protein Molecular Axis

**DOI:** 10.1002/kjm2.70076

**Published:** 2025-07-16

**Authors:** Yang Wang, You‐Xiang Hou, Li Xie, Yi‐La Xia, Yi‐Na Wang

**Affiliations:** ^1^ Department of Thoracic Surgery Affiliated Tumor Hospital of Xinjiang Medical University Urumqi China; ^2^ First Department of Gynecological Tumor Radiotherapy Affiliated Tumor Hospital of Xinjiang Medical University Urumqi China

**Keywords:** cervical cancer, long noncoding RNA TOB1‐AS1, microRNA‐27a‐3p, thioredoxin‐interacting protein

## Abstract

Cervical cancer (CC) remains a major global health concern, particularly due to its aggressive nature and limited treatment options in advanced stages. Long noncoding RNA (lncRNA) TOB1‐AS1 has been proposed as a tumor suppressor, yet its regulatory mechanism in CC remains unclear. This study aimed to elucidate the role of TOB1‐AS1 in CC progression through the miR‐27a‐3p/thioredoxin‐interacting protein (TXNIP) molecular axis. Functional gain‐ and loss‐of‐function assays were conducted to assess the effects of TOB1‐AS1, miR‐27a‐3p, and TXNIP on cell proliferation, invasion, migration, and apoptosis. RT‐qPCR, Western blotting, dual‐luciferase reporter assays, and in vivo xenograft models were used to validate interactions and phenotypic outcomes. TOB1‐AS1 was found to be downregulated in CC cells. Its overexpression suppressed proliferation, invasion, and migration, while enhancing apoptosis. Mechanistically, TOB1‐AS1 functioned as a competing endogenous RNA (ceRNA) by sponging miR‐27a‐3p, thereby restoring TXNIP expression. Modulating miR‐27a‐3p or TXNIP levels partially reversed the effects of TOB1‐AS1. In vivo, TOB1‐AS1 overexpression significantly inhibited tumor growth and altered miR‐27a‐3p and TXNIP expression profiles. These findings suggest that lncRNA TOB1‐AS1 acted as a ceRNA of miR‐27a‐3p to upregulate TXNIP, thereby suppressing CC cell proliferation, invasion and migration.

## 1 Introduction

Cervical cancer (CC) is the fourth most common malignancy among women worldwide. While advancements in screening and treatment have significantly reduced CC incidence and mortality in developed countries, the disease remains a major public health concern in developing regions, where prognosis is often poor due to limited access to healthcare and early detection programs [1, 2]. Treatment strategies for CC depend largely on disease stage and include neoadjuvant therapy, chemoradiotherapy, and combination chemotherapy [3]. However, recurrence and metastasis frequently lead to poor clinical outcomes, underscoring the urgent need for novel diagnostic and therapeutic strategies and a deeper understanding of the molecular mechanisms underlying CC progression.

Long noncoding RNAs (lncRNAs), defined as RNA transcripts longer than 200 nucleotides that lack protein‐coding potential, have emerged as crucial regulators of gene expression. Their functions span diverse mechanisms, including chromatin remodeling, protein binding, *cis*/*trans*‐transcriptional regulation, and serving as competing endogenous RNAs (ceRNAs) [4]. Increasing evidence highlights the involvement of lncRNAs in tumorigenesis and cancer progression [5]. For example, lncRNA SEMA3B‐AS1 has been identified as a tumor suppressor in triple‐negative breast cancer by targeting miR‐3940‐3p and regulating KLLN expression [6]. Similarly, WT1‐AS has been shown to suppress CC progression via modulation of specific oncogenic pathways [7].

Recent findings have reported reduced expression of lncRNA TOB1‐AS1 in CC, with evidence suggesting its tumor‐suppressive role through the modulation of miR‐27b [8]. However, the underlying mechanisms through which TOB1‐AS1 exerts its function in CC remain insufficiently explored. Given that lncRNAs can function as ceRNAs by sequestering miRNAs and thereby influencing mRNA targets, the TOB1‐AS1/miRNA/mRNA regulatory network may provide a novel insight into CC pathogenesis [9].

Interestingly, lncRNA TOB1‐AS1 has been shown to regulate tumor development through a ceRNA mechanism, acting as a molecular sponge for specific miRNAs to modulate gene expression [10]. In addition, aberrant expression of miRNAs has been implicated in altering the expression of tumor suppressors and oncogenes, thereby contributing to CC progression [11]. Among these, miR‐27a‐3p, located on chromosome 19, has been frequently reported to be dysregulated across several malignancies, including cervical, esophageal, and rectal cancers, where it promotes malignant behaviors such as proliferation, invasion, and metastasis [12].

In this study, bioinformatics analysis via the StarBase database identified miR‐27a‐3p as a potential downstream target of lncRNA TOB1‐AS1. This led us to hypothesize that TOB1‐AS1 may exert tumor‐suppressive functions in CC by modulating miR‐27a‐3p expression. In addition, thioredoxin‐interacting protein (TXNIP), also known as vitamin D3 upregulated protein‐1, plays a critical role in redox homeostasis [13]. TXNIP is involved in essential cellular processes such as energy metabolism, cell proliferation, and differentiation [14], and is widely recognized as a tumor suppressor that is frequently downregulated in a variety of cancers [13]. In the context of CC, TXNIP expression is notably decreased [15], and miR‐27a‐3p has been shown to directly target TXNIP through sequence‐specific binding [16].

Given these findings, we proposed that TOB1‐AS1 may inhibit CC cell proliferation, invasion, and migration through the miR‐27a‐3p/TXNIP axis. However, few studies to date have explored whether TOB1‐AS1 regulates this molecular cascade in CC. Therefore, the present research aimed to elucidate the mechanism by which TOB1‐AS1 influencing CC cell behavior through the miR‐27a‐3p/TXNIP axis, with the goal of identifying novel therapeutic targets and advancing effective treatment strategies for CC.

## 2 Materials and Methods

### 2.1 Ethics Statement

All animal procedures were reviewed and approved by the Animal Ethics Committee of Xinjiang Medical University and conducted in accordance with institutional and national guidelines.

### 2.2 Cell Culture

Human CC cell lines HeLa (CL‐0277), CaSki (CL‐0048), SiHa (CL‐0210), and C33A (CL‐0045) were purchased from Procell (Wuhan, China). Human endometrial epithelial cells (HEEpiC‐SV40; STM‐CL‐5397) were obtained from STEM RECELL (Shanghai, China). HeLa, CaSki, and HEEpiC‐SV40 cells were cultured in RPMI1640 medium (11875093, Gibco), while SiHa and C33A cells were maintained in Minimum Essential Medium (MEM; SH30024.02, HyClone). All media were supplemented with 10% fetal bovine serum (FBS; C0227, Ausgenex), 100 U/mL penicillin, and 100 mg/L streptomycin (15140122, Gibco). Cells were incubated at 37°C in a humidified atmosphere containing 5% CO_2_.

### 2.3 Cell Transfection and Grouping

Following the protocol of the manufacturer, HeLa cells were manipulated with the pcDNA3.1‐TOB1‐AS1 (oe‐TOB1‐AS1), pcDNA3.1‐negative control (oe‐NC), pcDNA3.1‐TXNIP (oe‐TXNIP), miR‐27a‐3p mimics, mimics‐NC, si‐TXNIP, and si‐NC (GenePharma, Shanghai, China). The transfections were achieved at a final concentration of 100 ng/μL utilizing Lipofectamine2000 (Invitrogen, Carlsbad, CA, USA). si‐TOB1‐AS1, si‐NC, si‐TXNIP, miR‐27a‐3p inhibitor, inhibitor‐NC, pcDNA3.1‐TXNIP (oe‐TXNIP), and pcDNA3.1‐NC (oe‐NC) (GenePharma) were transfected into CaSki cells at a final concentration of 100 ng/μL. Thereafter, cells were collected 48 h post transfections for subsequent experiments.

HeLa cells were grouped as hereafter: the Blank group (normally cultured cells), the oe‐NC group (cells delivered with oe‐NC), the oe‐AS1 group (cells delivered with oe‐TOB1‐AS1), the oe‐AS1 + mimics‐NC group (cells delivered with oe‐TOB1‐AS1 and mimics‐NC simultaneously), the oe‐AS1 + miR mimics group (cells delivered with oe‐TOB1‐AS1 and miR‐27a‐3p mimics simultaneously), the oe‐AS1 + si‐NC group (cells delivered with oe‐TOB1‐AS1 and si‐NC simultaneously), the oe‐AS1 + si‐TX group (cells simultaneously delivered with oe‐TOB1‐AS1 and si‐TXNIP), the oe‐AS1 + miR mimics + oe‐NC group (cells simultaneously treated with oe‐TOB1‐AS1, miR‐27a‐3p mimics and oe‐NC), the oe‐AS1 + miR mimics + oe‐TX group (cells delivered with oe‐TOB1‐AS1, miR‐27a‐3p mimics and oe‐TXNIP simultaneously). Three duplicates were conducted on each cell treatment independently.

CaSki cells were allocated into these groups: the Blank group (normally cultivated cells), the si‐NC group (cells delivered with si‐NC), the si‐AS1 group (cells delivered with si‐TOB1‐AS1), the si‐AS1 + inhibitor‐NC group (cells manifested with both si‐TOB1‐AS1 and miR‐27a‐3p inhibitor‐NC), the si‐AS1 + inhibitor group (cells treated simultaneously with si‐TOB1‐AS1 and miR‐27a‐3p inhibitor), the si‐AS1 + oe‐NC group (cells treated simultaneously with si‐TOB1‐AS1 and oe‐NC), the si‐AS1 + oe‐TX group (cells treated simultaneously with si‐TOB1‐AS1 and oe‐TXNIP), the si‐AS1 + inhibitor + si‐NC group (transfected simultaneously with si‐TOB1‐AS1, miR‐27a‐3p inhibitor and si‐NC), the si‐AS1 + inhibitor + si‐TX group (cells transfected simultaneously with si‐TOB1‐AS1, miR‐27a‐3p inhibitor and si‐TXNIP). Each cell treatment was independently replicated three times.

The lentiviral overexpression vectors and empty vectors were purchased from R&S Biotechnology (Shanghai, China). The lentiviral vectors were utilized to infect HeLa cells at a multiplicity of infection (MOI) of 4 to construct stable TOB1‐AS1‐overexpressing HeLa cells (LV‐TOB1‐AS1), with a viral titer of 1 × 10^8^ TU/mL [17].

### 2.4 Reverse Transcription Quantitative Polymerase Chain Reaction

Total RNA was extracted from cultured cells or mouse tumor tissues using TRIzol reagent (Thermo Fisher Scientific, Waltham, MA, USA). RNA concentration and purity were assessed with a spectrophotometer (Thermo Fisher Scientific). One microgram of RNA was reverse transcribed into complementary DNA (cDNA) using the PrimeScript RT Reagent Kit (Invitrogen, Carlsbad, CA, USA). Reverse transcription quantitative polymerase chain reaction (RT‐qPCR) was performed on an ABI ViiA7DX System (Applied Biosystems, Foster City, CA, USA). Gene expression levels were normalized to U6 (for miRNAs) and GAPDH (for mRNAs) using the 2−^ΔΔ*C*t^ method. Primer sequences used are listed in Table 1.

**TABLE 1:** Primer sequence.

Gene	Forward 5′–3′	Reverse 5′–3′
TOB1‐AS1	GCCAGGCCTAGAAGCTTTTG	TCTTCCCACCCCTTCTCCTA
miR‐27a‐3p	TGCGGTTCACAGTGGCTAAG	CTCAACTGGTGTCGTGGA
TXNIP	TGTGTGAAGTTACTCGTGTCAAA	GCAGGTACTCCGAAGTCTGT
U6	CTCGCTTCGGCAGCACA	AACGCTTCACGAATTTGCGT
GAPDH	CGACTTATACATGGCCTTA	TTCCGATCACTGTTGGAAT

### 2.5 Western Blot Analysis

Proteins were extracted using RIPA lysis buffer (AR0107, Boster, Wuhan, Hubei, China) and quantified with a bicinchoninic acid protein assay kit (AR1189, Boster). Equal amounts of protein (20 μg per lane) were mixed with loading buffer, denatured at 95°C for 5 min, separated by sodium dodecyl sulfate‐polyacrylamide gel electrophoresis, and transferred to a polyvinylidene fluoride membrane. Membranes were blocked with 3% BSA (AR0184, Boster) for 2 h at room temperature and incubated overnight at 4°C with primary antibodies (listed in Table 2). After washing, membranes were incubated with HRP‐conjugated goat anti‐rabbit immunoglobulin G H&L (HRP) secondary antibody for 1 h at room temperature. Protein bands were visualized using an enhanced chemiluminescence kit (AR1191, Boster) and quantified using Image Pro Plus 6.0 software (Media Cybernetics, Bethesda, MD, USA). GAPDH served as a loading control. Experiments were independently repeated three times.

**TABLE 2:** Antibodies for Western blot.

Antibodies	Item no. and company	Dilution ratio
TXNIP	Ab188865, Abcam	1/1000
GAPDH	Ab181602, Abcam	1/1000
IgG H&L (HRP)	Ab6721, Abcam	1/2000

### 2.6 Cell Counting Kit‐8 Assay

Cell viability was assessed using the Cell Counting Kit‐8 (CCK‐8) assay (CA1210, Solarbio, Beijing, China). Cells were seeded into 96‐well plates at a density of 5 × 10^3^ cells/well and cultured for 0, 12, 24, and 48 h. At each time point, 100 μL of CCK‐8 reagent was added to each well, followed by a 2‐h incubation at 37°C. Absorbance was measured at 450 nm using a microplate reader (Thermo Fisher Scientific).

### 2.7 Transwell Assay

Cell invasion and migration abilities were assessed using Transwell chambers (Corning, NY, USA). For invasion assays, the upper chambers were pre‐coated with Matrigel (BD Biosciences, San Jose, CA, USA); for migration assays, chambers were used without Matrigel. Cells were serum‐starved for 24 h, then resuspended in medium containing 1% FBS. A total of 1 × 10^5^ cells were seeded in the upper chamber, while 600 μL of medium supplemented with 10% FBS was added to the lower chamber. After a 24‐h incubation at 37°C, non‐migrated/invaded cells were removed with a cotton swab. Remaining cells on the lower membrane surface were fixed with 4% paraformaldehyde (158127, MedChemExpress, Monmouth Junction, NJ, USA) and stained with hematoxylin (517‐28‐2, Macklin). Cells were counted in five randomly selected fields under a microscope.

### 2.8 Cell Scratch Assay

Cell migration was assessed via a wound healing (scratch) assay. Cells were seeded into six‐well plates at a density of 1 × 10^4^ cells/well and cultured until 80%–90% confluence. A linear scratch was made through the monolayer using a sterile P‐200 pipette tip. After removing detached cells with phosphate‐buffered saline (PBS), serum‐free Dulbecco's modified Eagle medium (DMEM) was added. Wound closure was imaged at 0 h and 24 h using an inverted microscope (TS100, Nikon, Tokyo, Japan). The migration rate was calculated using the formula:

Cell migration rate = wound width (24 h)/wound width (0 h) [18].

### 2.9 Flow Cytometry

Apoptosis was evaluated using flow cytometry. Cells were collected and washed three times with cold PBS. After resuspension in 0.3 mL PBS, cells were fixed in 0.7 mL of cold absolute ethanol at −20°C for 24 h. Fixed cells were centrifuged at 1000 rpm for 15 min and washed twice with PBS. The cell pellet was resuspended in 120 μL of RNase A solution (200 μg/mL) and incubated at 37°C for 30 min. Apoptosis was detected using Annexin V‐FITC (BD Biosciences) and propidium iodide (PI) staining (Beyotime, Shanghai, China); followed by analysis on a flow cytometer [19].

### 2.10 Dual‐Luciferase Reporter Assay

The target binding sites of miR‐27a‐3p with lncRNA TOB1‐AS1 and TXNIP were predicted by the Starbase database (https://rnasysu.com/encori/), respectively. The synthesized gene fragments of TOB1‐AS1 and TXNIP 3′‐UTR were introduced into the pMIR‐reporter (KL‐ZL‐1015‐01, Ke Lei Biotechnology Co. Ltd., Shanghai, China) via the endonuclease sites *Bam*H1 and *Eco*RI. Mutation sites for complementary sequences of seed sequences were designed on the TOB1‐AS1 and TXNIP wild‐type (WT), followed by detachment with restriction endonuclease and insertion of the target fragments into the pMIR‐reporter plasmid via the T4 DNA ligase. The identified correct luciferase reporter plasmids WT and mutant (MUT) were co‐transfected with NC mimics and miR‐27a‐3p mimics into HeLa and CaSki cells. Cells were lysed and detected by luciferase detection kit (HY K1013, MedChemExpress) 48 h subsequent to transfection.

### 2.11 Xenograft Tumor Model in Nude Mice

Female BALB/c nude mice (4 weeks old, *n* = 18) were obtained from TECON Biopharmaceutical Co. Ltd. (Urumqi, Xinjiang, China, Animal License number: SYXK [Xinjiang] 2021‐0005). Mice were housed under standard conditions (20°C–26°C, 12 h light/dark cycle, ad libitum access to food and water).

HeLa cells stably overexpressing TOB1‐AS1 (1 × 10^7^) were subcutaneously injected into the right flank of mice. Tumor volume was measured every 5 days using calipers and calculated using the formula: (*a* × *b* ^2^)/2, where a and b denote the longest longitudinal and transverse diameters, respectively [20]. After 4 weeks, mice were sacrificed by intraperitoneal injection of 150 mg/kg sodium pentobarbital (Sigma‐Aldrich, St. Louis, MO, USA). Tumors were excised, weighed, and partially fixed in 4% paraformaldehyde for immunohistochemistry (IHC). Remaining tissues were snap‐frozen for RNA extraction and RT‐qPCR.

Mice were randomly divided into three groups (*n* = 6 per group): Control group (injected with untreated HeLa cells), LV‐NC (injected with HeLa cells transfected with negative control lentiviral vector), LV‐TOB1‐AS1 group (injected with HeLa cells transfected with TOB1‐AS1‐overexpressing lentivirus).

### 2.12 IHC

Paraffin‐embedded tumor tissue sections from xenograft mice were first baked overnight at 60°C, followed by additional incubation in a constant‐temperature oven at 45°C for 3 h. After dewaxing in xylene and rehydration through graded ethanol solutions, antigen retrieval was performed using citrate buffer (PR30001, Proteintech, Wuhan, Hubei, China). The buffer was brought to a boil and then allowed to cool for 5–10 min, with this heating–cooling cycle repeated three times before cooling to room temperature. Sections were washed three times in Tris‐buffered saline (TBS; ST667, Beyotime) and incubated with 3% hydrogen peroxide at room temperature for 10 min to quench endogenous peroxidase activity, followed by three additional TBS washes. After blocking with 5% goat serum (C0265, Beyotime) in a humidified chamber at 37°C for 1 h, the serum was removed, and sections were incubated overnight at 4°C with the following primary antibodies: rabbit anti‐Ki‐67 (ab15580, Abcam, Cambridge, UK), rabbit anti‐TXNIP (ab188865, Abcam). The following day, slides were returned to room temperature and washed three times with TBS. A horseradish peroxidase (HRP)‐conjugated anti‐rabbit antibody (ab6721, Abcam) was applied at 37°C for 15 min in a humidified chamber. After washing, color development was achieved using diaminobenzidine (DAB; P0202, Beyotime) for 15 min at 37°C. Sections were then counterstained with hematoxylin, dehydrated, and mounted. Images were captured under a light microscope (Olympus, Tokyo, Japan). The percentage of positively stained cells was quantified in five random fields per section, and the mean value was used for analysis.

### 2.13 Statistical Analysis

All statistical analyses and data visualization were performed using GraphPad Prism version 8.01 (GraphPad Software, San Diego, CA, USA). The normality of data distribution was assessed using the Kolmogorov–Smirnov test. For data following a normal distribution, results were expressed as mean ± standard deviation. Comparisons between two groups were analyzed using the independent sample *t*‐test, while multigroup data comparisons were executed by one‐way analysis of variance (ANOVA), followed by Tukey's multiple comparison test. A two‐tailed *p* < 0.05 was considered statistically significant.

## 3 Results

### 3.1 lncRNA TOB1‐AS1 Inhibited CC Cell Proliferation, Migration, and Invasion

**FIGURE 1:**
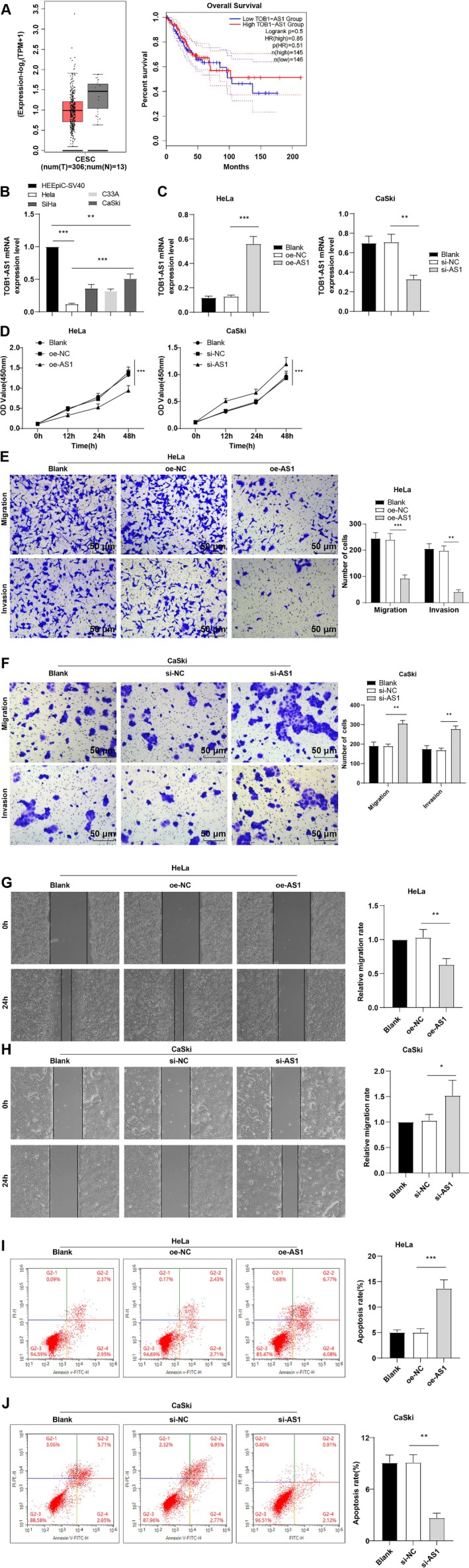
Poor expression of lncRNA TOB1‐AS1 was observed in CC cells, and its overexpression inhibited cell migration, proliferation and invasion in CC. (A) Relationship between TOB1‐AS1 expression in CC and overall survival was analyzed using the GEPIA2 database (http://gepia2.cancer‐pku.cn/#index). (B, C) RT‐qPCR to determine TOB1‐AS1 mRNA expression. (D) CCK‐8 assay to assess cell proliferation. (E, F) Cell invasion and migration were evaluated by Transwell assay. (G, H) Cell migration was assessed by cell scratch assay. (I, J) Cell apoptosis was evaluated by flow cytometry. The cell experiments were repeated independently thrice, with the data represented as mean ± standard deviation. One‐way ANOVA was conducted to compare the data among multiple groups, with Tukey's multiple comparison test used for post hoc test. *p < 0.05, **p < 0.01, ***p < 0.001.

Analysis of the GEPIA2 database (http://gepia2.cancer‐pku.cn/#index) revealed that TOB1‐AS1 expression was significantly downregulated in CC tissues, and low expression correlated with shorter overall survival (Figure 1A). RT‐qPCR confirmed that TOB1‐AS1 was markedly reduced in CC cell lines (HeLa, SiHa, C33A, and CaSki), with the lowest levels observed in HeLa cells (*p* < 0.001, Figure 1B). To explore its function, TOB1‐AS1 was overexpressed in HeLa cells and silenced in CaSki cells via transfection with oe‐TOB1‐AS1 or si‐TOB1‐AS1, respectively. RT‐qPCR validated effective modulation of TOB1‐AS1 expression (*p* < 0.01, Figure 1C). CCK‐8 assays revealed that TOB1‐AS1 overexpression suppressed HeLa cell proliferation, while its knockdown promoted proliferation in CaSki cells (*p* < 0.01, Figure 1D). Similarly, Transwell and scratch assays demonstrated reduced invasion and migration in oe‐TOB1‐AS1‐transfected cells and increased motility in si‐TOB1‐AS1‐transfected cells (all *p* < 0.05, Figure 1E–H). Flow cytometry further showed enhanced apoptosis in the oe‐TOB1‐AS1 group and reduced apoptosis in the si‐TOB1‐AS1 group (*p* < 0.001, Figure 1I,J). Together, these findings indicated that TOB1‐AS1 is downregulated in CC, and its restoration suppresses malignant behaviors.

### 3.2 TOB1‐AS1 Suppressed CC Progression by Downregulating miR‐27a‐3p

**FIGURE 2:**
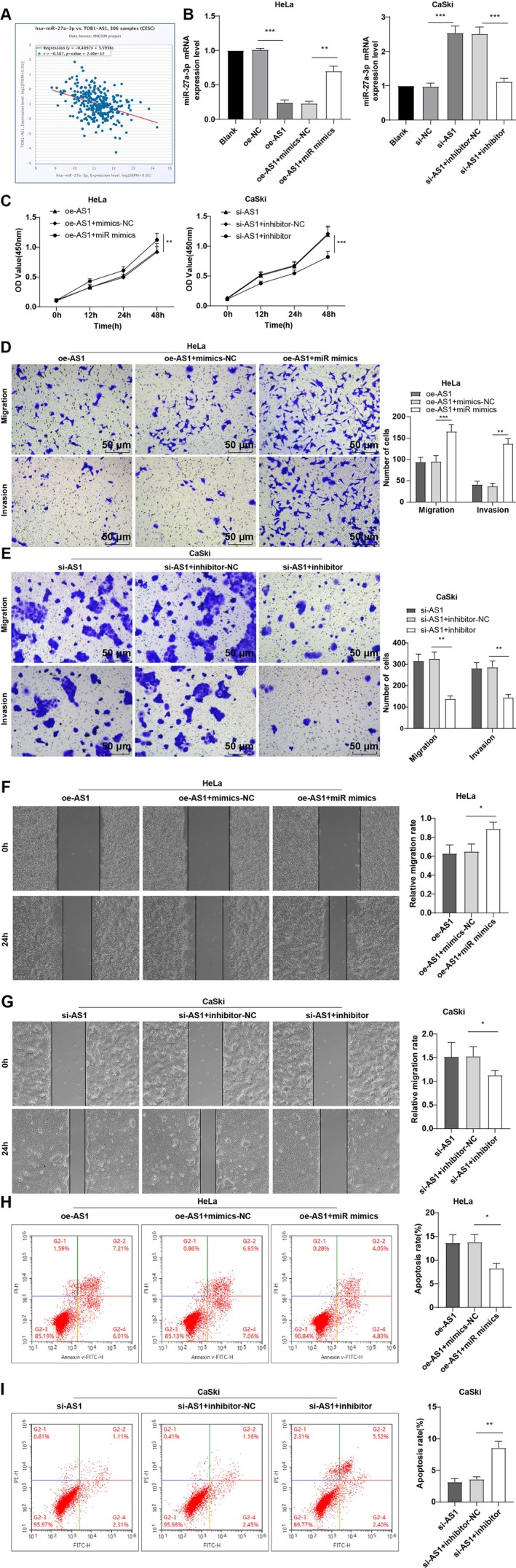
lncRNA TOB1‐AS1 limited cell invasion, migration and proliferation in CC by downregulating miR‐27a‐3p. (A) The correlation between TOB1‐AS1 and miR‐27a‐3p in CC was analyzed through StarBase prediction. (B) miR‐27a‐3p mRNA expression was determined by RT‐qPCR. (C) Cell proliferation was assessed by CCK‐8 assay. (D, E) Determination of cell migration and invasion by Transwell assay. (F, G) Cell scratch test to evaluate cell migration. (H, I) Cell apoptosis was evaluated by flow cytometry. Three repetitions were guaranteed independently in cell experiments, with the data expressed as mean ± standard deviation. Data comparisons were performed among multiple groups by one‐way ANOVA, followed by Tukey's multiple comparison test. *p < 0.05, **p < 0.01, ***p < 0.001.

Given prior evidence of miR‐27a‐3p promoting CC aggressiveness [12], we investigated its relationship with TOB1‐AS1. StarBase analysis predicted an inverse correlation between TOB1‐AS1 and miR‐27a‐3p in CC (Figure 2A). RT‐qPCR showed that miR‐27a‐3p expression was significantly downregulated by TOB1‐AS1 overexpression and upregulated following its knockdown (*p* < 0.001, Figure 2B). Co‐transfection experiments demonstrated that miR‐27a‐3p mimics reversed the inhibitory effects of TOB1‐AS1 overexpression on HeLa cell proliferation, migration, and invasion while reducing apoptosis. Conversely, miR‐27a‐3p inhibition reversed the oncogenic effects of TOB1‐AS1 knockdown in CaSki cells, suppressing proliferation, invasion, and migration while enhancing apoptosis (all *p* < 0.05, Figure 2C–I). These results suggest that TOB1‐AS1 exerts its tumor‐suppressive effects by targeting miR‐27a‐3p.

### 3.3 TOB1‐AS1 Upregulated TXNIP by Sponging miR‐27a‐3p

**FIGURE 3:**
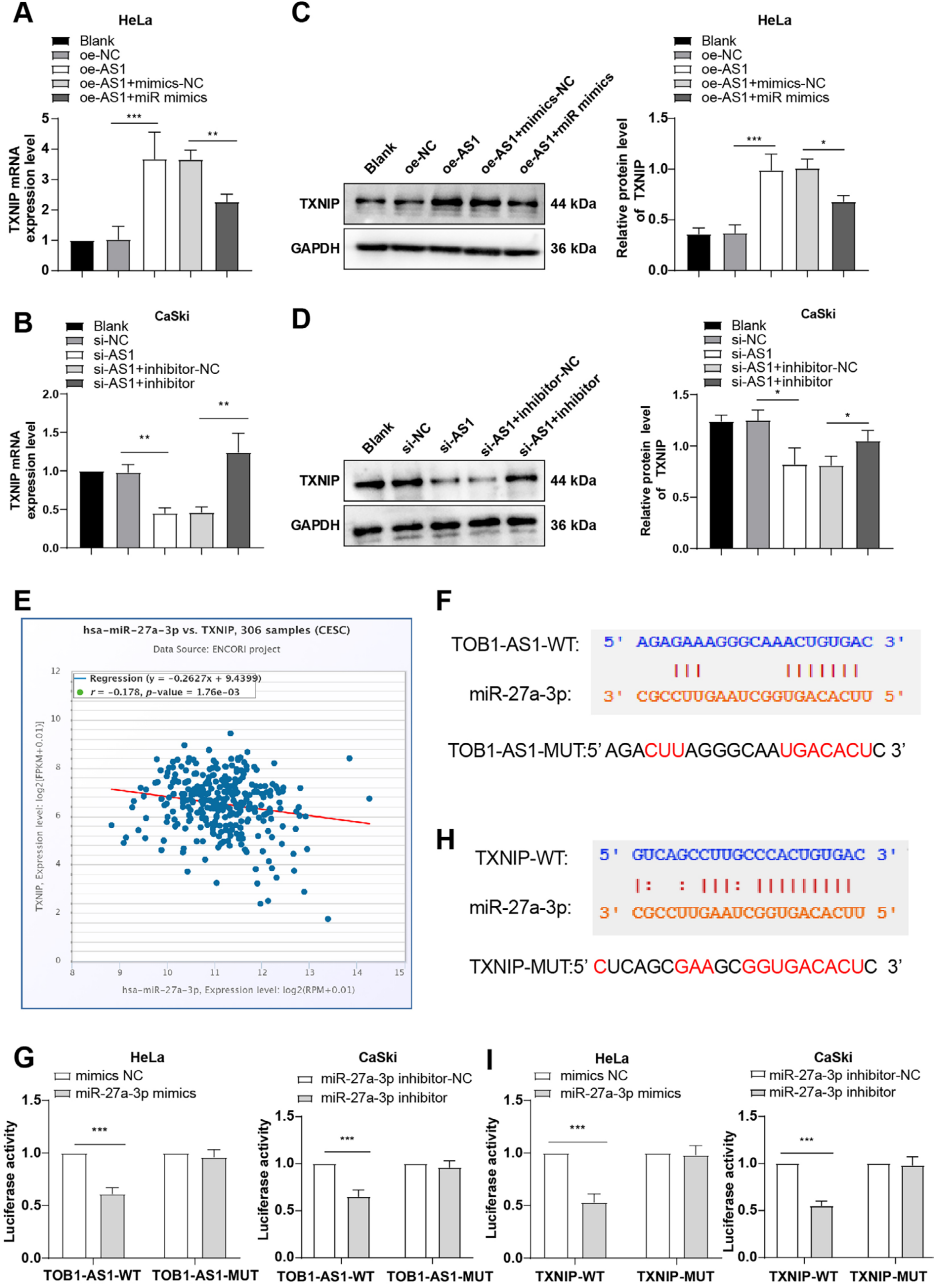
lncRNA TOB1‐AS1 functioned as a ceRNA of miR‐27a‐3p to control TXNIP expression. (A, B) TXNIP mRNA expression was determined by RT‐qPCR. (C, D) TXNIP protein expression was measured by Western blot. Starbase database to predict (E) the correlation between miR‐27a‐3p and TXNIP in CC and the target binding sites between (F) lncRNA TOB1‐AS1 and miR‐27a‐3p, and between (H) miR‐27a‐3p and TXNIP. Dual‐luciferase assay to verify the target binding relationships between (G) lncRNA TOB1‐AS1 and miR‐27a‐3p, and between (I) miR‐27a‐3p and TXNIP. The cellular experiments were repeated thrice independently. The data were represented by mean ± standard deviation, with one‐way ANOVA used for comparisons of the data among multiple groups and Tukey's test used for post hoc test. *p < 0.05, **p < 0.01, ***p < 0.001.

Western blot and RT‐qPCR revealed that TXNIP expression was upregulated following TOB1‐AS1 overexpression and suppressed by miR‐27a‐3p mimics (all *p* < 0.05, Figure 3A,C). Conversely, TOB1‐AS1 knockdown reduced TXNIP levels, which were restored by miR‐27a‐3p inhibitors (all *p* < 0.05, Figure 3B,D). StarBase predicted that miR‐27a‐3p directly targets both TOB1‐AS1 and TXNIP (Figure 3E,F,H). Dual‐luciferase reporter assays confirmed direct interactions between miR‐27a‐3p and the 3′‐UTRs of TOB1‐AS1 and TXNIP (*p* < 0.001, Figure 3G–I). These findings indicate that TOB1‐AS1 functions as a ceRNA to sponge miR‐27a‐3p and upregulate TXNIP expression. Notably, RT‐qPCR showed that miR‐27a‐3p modulates TOB1‐AS1 levels in return, suggesting a feedback mechanism (Figure S1). These results suggested that miR‐27a‐3p in turn regulated TOB1‐AS1 expression, but the regulatory mechanism remained to be further elucidated.

### 3.4 TXNIP Modulation Partially Reversed the Regulatory Effects of TOB1‐AS1 on CC Cell Malignancy

**FIGURE 4:**
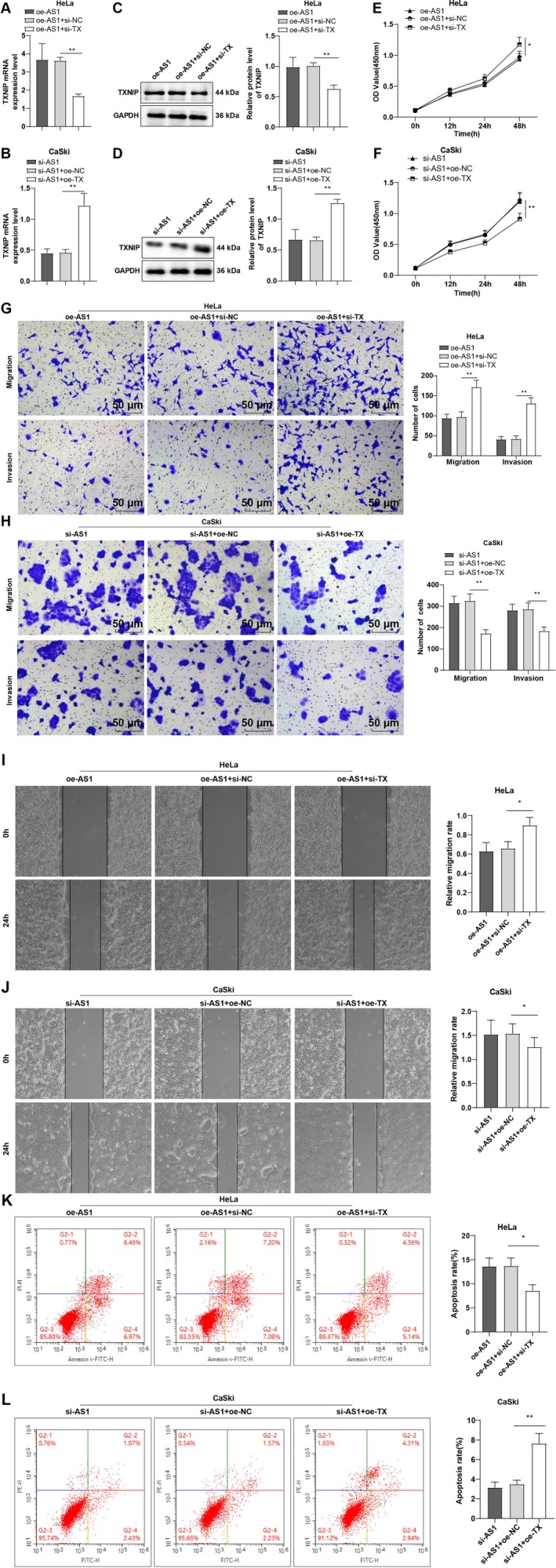
Modulating TXNIP partially averted the impacts of lncRNA TOB1‐AS1CC on cell proliferation, migration and invasion. (A, B) TXNIP mRNA expression was measured by RT‐qPCR. (C, D) TXNIP protein expression was determined by Western blot. (E, F) Cell proliferation was assessed by CCK‐8 assay. (G, H) Transwell assay to evaluate cell invasion and migration. (I, J) Cell migration was evaluated by cell scratch test. (K, L) Apoptosis was assessed by flow cytometry. The cellular experiments were repeated thrice independently. The data were represented by mean ± standard deviation, with one‐way ANOVA adopted for comparisons of data among multiple groups and Tukey's test adopted for post hoc tests. *p < 0.05, **p < 0.01.

To further elucidate whether TXNIP mediates the tumor‐suppressive role of lncRNA TOB1‐AS1 in CC cells, rescue assays were performed. HeLa cells were co‐transfected with oe‐TOB1‐AS1 and si‐TXNIP, while CaSki cells were co‐transfected with si‐TOB1‐AS1 and oe‐TXNIP. In HeLa cells, si‐TXNIP reversed the effect of TOB1‐AS1 overexpression by downregulating TXNIP expression (all *p* < 0.01, Figure 4A,C), enhancing proliferation, invasion, and migration, and reducing apoptosis (all *p* < 0.05, Figure 4E–K). Conversely, overexpression of TXNIP in TOB1‐AS1‐silenced CaSki cells upregulated TXNIP expression (all *p* < 0.01, Figure 4B,D), suppressed proliferation, invasion, and migration, and promoted apoptosis (all *p* < 0.05, Figure 4F–L). These findings indicate that TXNIP is a critical downstream effector of TOB1‐AS1 and partially mediates its inhibitory effects on CC cell malignant behaviors.

### 3.5 TOB1‐AS1 Suppressed CC Cell Malignancy Through the miR‐27a‐3p/TXNIP Molecular Axis

**FIGURE 5:**
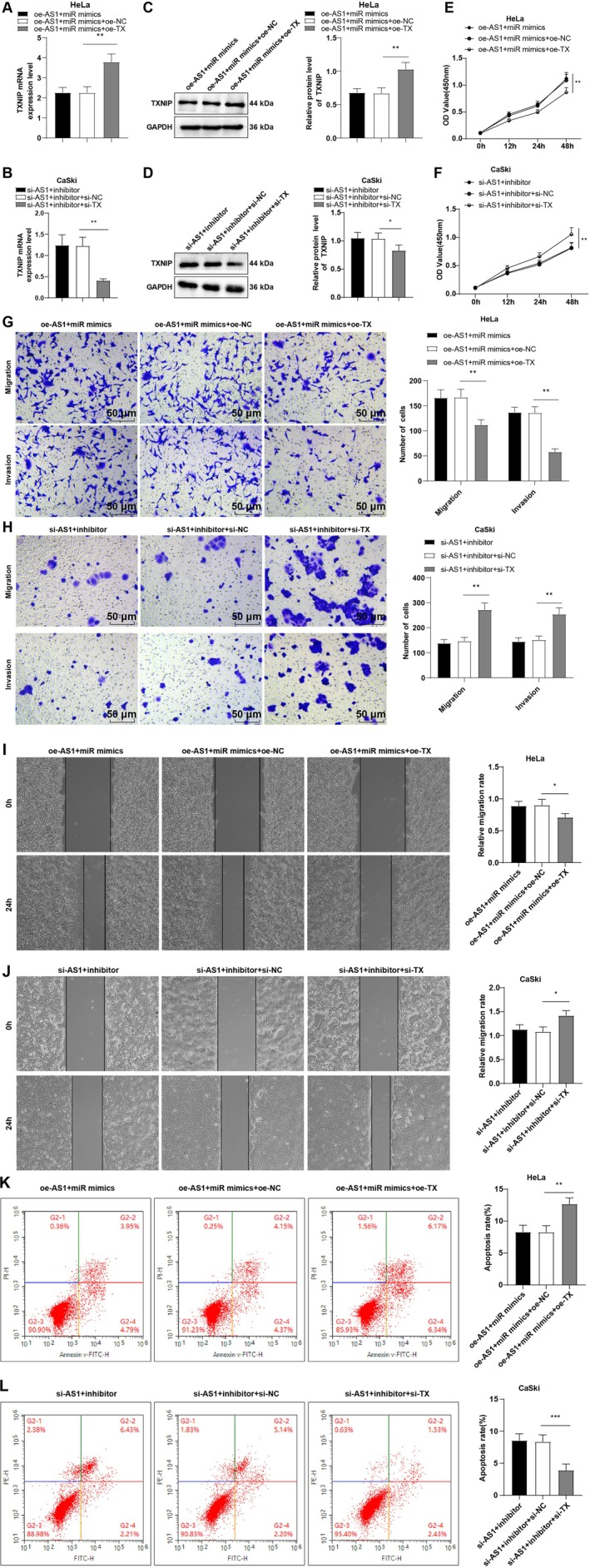
lncRNA TOB1‐AS1 impeded CC cell invasion, proliferation and migration via the miR‐27a‐3p/TXNIP axis. (A, B) RT‐qPCR to determine TXNIP mRNA expression. (C, D) Western blot to measure the expression of TXNIP protein. (E, F) CCK‐8 assay to evaluate cell proliferation. (G, H) Transwell assay to assess cell migration and invasion. (I, J) Cell scratch test to determine cell migration. (K, L) Apoptosis was evaluated by flow cytometry. The cellular experiments were independently repeated three times, with the data depicted as mean ± standard deviation. One‐way ANOVA was used to compare the data among multiple groups, followed by Tukey's test. *p < 0.05, **p < 0.01.

To further confirm that TOB1‐AS1 regulates CC cell progression via the miR‐27a‐3p/TXNIP pathway, HeLa cells were co‐transfected with oe‐TOB1‐AS1, miR‐27a‐3p mimics, and oe‐TXNIP, and CaSki cells were co‐transfected with si‐TOB1‐AS1, miR‐27a‐3p inhibitor, and si‐TXNIP. In HeLa cells, TXNIP expression was restored in the oe‐AS1 + miR mimics + oe‐TX group compared to the oe‐AS1 + miR mimics + oe‐NC group (all *p* < 0.01, Figure 5A,C), with accompanying reductions in cell proliferation, invasion, and migration, and enhanced apoptosis (all *p* < 0.05, Figure 5E,K). In contrast, CaSki cells in the si‐AS1 + inhibitor + si‐TX group exhibited decreased TXNIP expression (all *p* < 0.05, Figure 5B,D), increased malignant phenotypes, and reduced apoptosis compared to the si‐AS1 + inhibitor + si‐NC group (all *p* < 0.05, Figure 5F,L). These data confirm that TOB1‐AS1 suppresses CC cell aggressiveness through the miR‐27a‐3p/TXNIP regulatory axis.

### 3.6 TOB1‐AS1 Overexpression Inhibited CC Tumor Growth In Vivo

**FIGURE 6:**
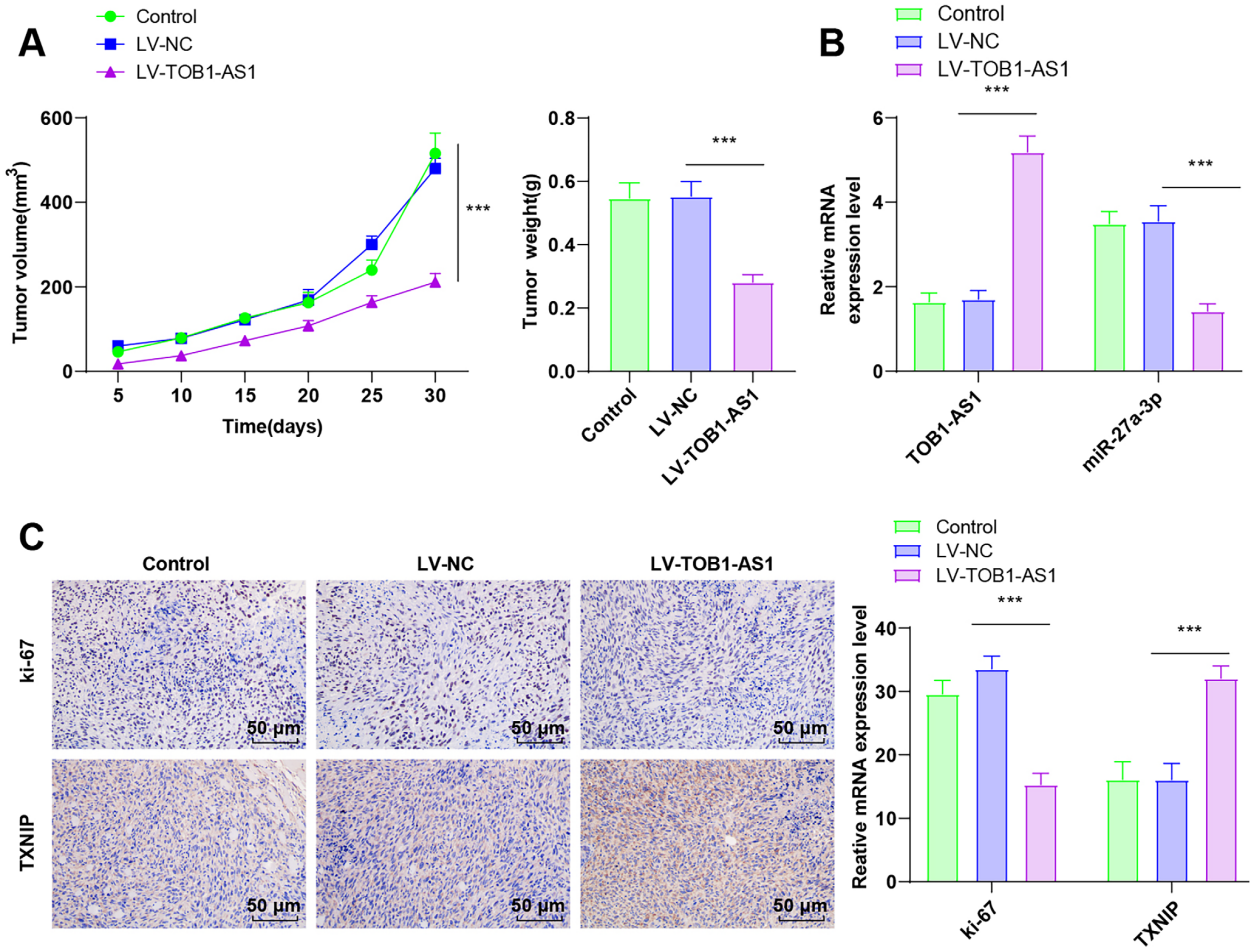
The effect of overexpression of TOB1‐AS1 on tumor growth in mice was verified in vivo. (A) Tumor volume and weight in mice, *n* = 6. (B) RT‐qPCR to determine TOB1‐AS1 and miR‐27a‐3p expression in mouse tumor tissues, *n* = 6. (C) Immunohistochemistry to determine Ki‐67 and TXNIP expression in mouse tumor tissues, *n* = 3. Data were depicted as mean ± standard deviation, with one‐way ANOVA applied for intergroup comparisons, and Tukey's multiple comparison test for the post hoc analysis. ***p < 0.001.

To validate the in vitro findings in vivo, a xenograft mouse model was constructed using HeLa cells stably overexpressing TOB1‐AS1. Compared to the LV‐NC group, mice in the LV‐TOB1‐AS1 group exhibited significantly smaller tumor volumes and weights (all *p* < 0.001, Figure 6A). RT‐qPCR analysis of tumor tissues showed upregulated TOB1‐AS1 and downregulated miR‐27a‐3p in the LV‐TOB1‐AS1 group (all *p* < 0.001, Figure 6B). Immunohistochemical staining revealed a reduced percentage of Ki‐67‐positive cells and enhanced TXNIP expression in tumors from LV‐TOB1‐AS1 mice (all *p* < 0.001, Figure 6C). The results indicated that TOB1‐AS1 overexpression decreased miR‐27a‐3p expression and increased TXNIP expression in tumor tissues of tumor‐bearing mice and inhibited tumor growth in vivo.

## 4 Discussion

CC remains a major global health challenge, particularly in developing regions, where chronic HPV infection is a leading cause of disease progression [7]. While early‐stage CC can be managed effectively through surgery and radiotherapy, treatment options for metastatic or recurrent disease are limited and often ineffective, underscoring the urgent need for novel molecular targets and therapeutic strategies [21]. Recent evidence has highlighted the pivotal role of lncRNAs in regulating the biological behaviors of CC cells [22]. However, the functional implications of lncRNA TOB1‐AS1 in CC cells remain poorly understood. This study demonstrates that lncRNA TOB1‐AS1 suppresses CC cell malignancy by modulating the miR‐27a‐3p/TXNIP axis, offering new insights into its therapeutic potential.

lncRNAs have emerged as critical regulators in CC progression, as exemplified by well‐characterized oncogenic and tumor‐suppressive lncRNAs such as HOTAIR and PVT1 [23]. Previous findings suggest that TOB1‐AS1 is downregulated in CC and may serve as a tumor suppressor [8]. In the present study, gain‐ and loss‐of‐function experiments showed that overexpression of TOB1‐AS1 significantly reduced CC cell proliferation, invasion, and migration, while promoting apoptosis. Conversely, TOB1‐AS1 knockdown led to enhanced malignant phenotypes, though its effect on apoptosis was not statistically significant. These findings are in line with prior studies, such as the report by Jiang et al., which demonstrated that TOB1‐AS1 inhibits migration and proliferation in gastric carcinoma via the miR‐23a/NEU1 axis [10], and another showing its tumor‐suppressive role in esophageal cancer [24]. Taken together, our data support that TOB1‐AS1 acts as a potent suppressor of CC progression.

miR‐27a‐3p is well‐documented as an oncogenic miRNA in various cancers, including CC, where it enhances proliferation, invasion, and migration [12]. It also functions as a downstream effector in several tumor‐suppressive lncRNA axes, such as RMST/miR‐27a‐3p/RXRα in colorectal cancer [25], and LINC01089/miR‐27a‐3p/BTG2 in CC [26]. Our findings revealed that TOB1‐AS1 overexpression led to downregulation of miR‐27a‐3p, while TOB1‐AS1 knockdown upregulated miR‐27a‐3p. Furthermore, the malignant phenotypes regulated by TOB1‐AS1 were partially reversed by altering miR‐27a‐3p levels, indicating a functional interaction.

Interestingly, our data suggest that miR‐27a‐3p may also regulate TOB1‐AS1, forming a potential feedback loop. Previous research indicates that TOB1‐AS1 can regulate transcriptional regulatory factors such as ATF3, which are known to influence miRNA transcription [24]. miR‐27a‐3p itself has been shown to target transcription factors like ATF3 [27], and TOB1 may also be targeted by other miRNAs such as miR‐92a‐3p [28]. These findings raise the possibility of a reciprocal regulatory relationship between TOB1‐AS1 and miR‐27a‐3p, mediated through transcriptional feedback or pathway modulation, including Wnt/β‐catenin or MAPK/ERK pathways [10, 29, 30, 31, 32]. In our in vivo models, we confirmed that TOB1‐AS1 overexpression suppressed miR‐27a‐3p expression in tumor tissues. Together, these data establish that TOB1‐AS1 exerts tumor‐suppressive effects by inhibiting miR‐27a‐3p, which in turn negatively regulates TOB1‐AS1, suggesting a feedback regulatory loop between these two noncoding RNAs.

TOB1‐AS1 has previously been reported to inhibit invasion and migration in non–small cell lung cancer via a ceRNA mechanism. In general, lncRNA can act as a ceRNA by competitively binding mRNAs, thereby modulating downstream mRNA targets [33]. In this context, TXNIP, a known tumor suppressor, is often downregulated in CC [14, 15]. Our study demonstrated that TXNIP expression was significantly upregulated in CC cells following TOB1‐AS1 overexpression, while it decreased upon miR‐27a‐3p expression. Conversely, TXNIP levels declined after TOB1‐AS1 knockdown but increased when miR‐27a‐3p was inhibited. StarBase predictions and dual‐luciferase assays confirmed direct binding interactions between miR‐27a‐3p and both TOB1‐AS1 and TXNIP, supporting the hypothesis that TOB1‐AS1 regulates TXNIP via a ceRNA mechanism.

Our findings align with previous reports, such as the study showing that STARD7‐AS1 suppresses CC cell proliferation through the miR‐31‐5p/TXNIP axis [34], and another demonstrating miR‐27a‐3p's targeting of TXNIP in cancer [16]. For the first time, we provide direct evidence that TOB1‐AS1 functions as a ceRNA for miR‐27a‐3p, thereby regulating TXNIP expression in CC.

To validate this regulatory axis functionally, we demonstrated that silencing TXNIP partially averted the effects of TOB1‐AS1 overexpression, while TXNIP mitigated the oncogenic effects of TOB1‐AS1 knockdown. Furthermore, co‐transfection experiments confirmed that TOB1‐AS1 limited the malignant phenotype of CC cells via the miR‐27a‐3p/TXNIP axis. These findings are corroborated by prior reports showing that TXNIP suppresses CC cell proliferation and migration in HeLa cells and promotes apoptosis [35]. TOB1‐AS1 often epigenetically silenced in tumors, also exerts anti‐tumor activity through sponging of other miRNAs like miR‐27b [8]. Similarly, LINC01089 inhibits gastric cancer cell growth by targeting miR‐27a‐3p and enhancing TET1 expression [36], and STARD7‐AS1 has been reported to modulate cell proliferation and autophagy through the miR‐31‐5p/TXNIP [34].

In summary, this study identifies TOB1‐AS1 as a negative regulator of CC progression through the miR‐27a‐3p/TXNIP molecular axis. This axis represents a promising avenue for therapeutic targeting in CC. However, this study has several limitations. We focused solely on the TOB1‐AS1/miR‐27a‐3p/TXNIP regulatory network without validating the mechanism in clinical tissue samples. In addition, we did not explore the downstream signaling cascades activated by TXNIP.

Future research will expand on these findings by investigating other miRNAs and pathways regulated by TOB1‐AS1, including IL‐6/STAT3, miR‐599/TLR4, and miR‐191‐5p/TRAF3. The role of TXNIP in ferroptosis, as suggested by its status as a ferroptosis‐related biomarker in cervical squamous cell carcinoma [37], also warrants investigation. Moreover, the crosstalk between TXNIP and oncogenic signaling pathways such as MAPK and PI3K/Akt [38, 39, 40], as well as its role in immunoregulation via the JAK–STAT pathway [39], should be explored further. In vivo animal models and clinical sample validation will be crucial in translating these molecular findings into diagnostic and therapeutic advances.

## Ethics Statement

All animal procedures were reviewed and approved by the Animal Ethics Committee of Xinjiang Medical University and conducted in accordance with institutional and national guidelines.

## Conflicts of Interest

The authors declare no conflicts of interest.

## Supporting information


**Figure S1.** lncRNA TOB1‐AS1 was regulated by miR‐27a‐3p. The mRNA expression of TOB1‐AS1 was determined by RT‐qPCR. The cell experiments were independently repeated three times, and data were presented as mean ± standard deviation. Data comparisons among multiple groups were analyzed using one‐way ANOVA, and the post hoc analysis using Tukey’s multiple comparison test. *p < 0.05, **p < 0.01, ***p < 0.001.

**Data S1.** Supporting Information.

## Data Availability

The findings of this study are available from the corresponding author upon reasonable request.
