# Supplementary figures and images for: Long Noncoding RNA TOB1‐AS1 Represses Cervical Cancer Cell Proliferation, Invasion, and Migration via the MicroRNA‐27a‐3p/Thioredoxin‐Interacting Protein Molecular Axis
Source: Kaohsiung J Med Sci. 2025 Jul 16;41(11):e70076. doi: 10.1002/kjm2.70076 (PMC12622466; doi:10.1002/kjm2.70076)

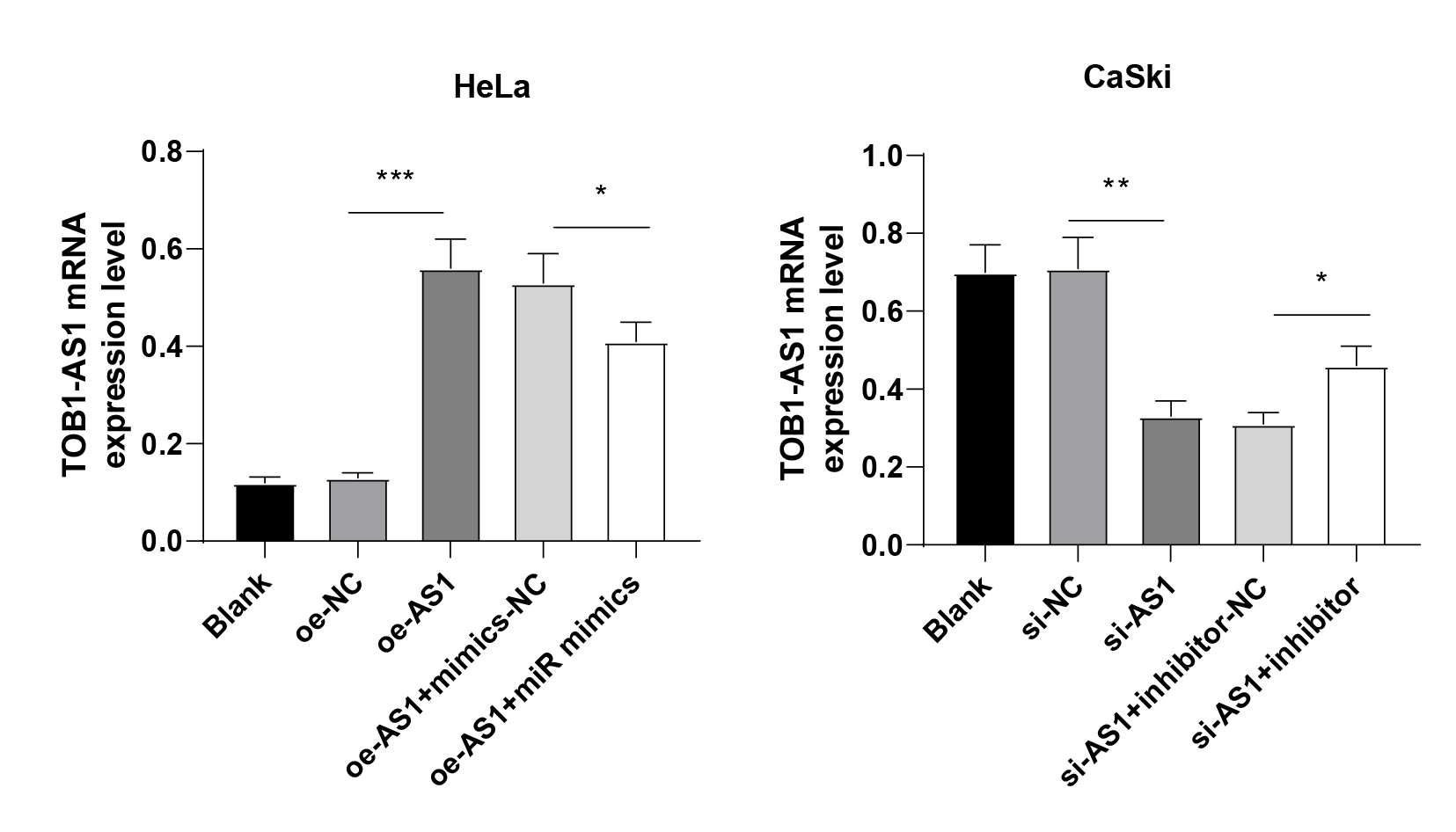

Supplement: Supplementary file 1 — Figure S1. lncRNA TOB1‐AS1 was regulated by miR‐27a‐3p. The mRNA expression of TOB1‐AS1 was determined by RT‐qPCR. The cell experiments were independently repeated three times, and data were presented as mean ± standard deviation. Data comparisons among multiple groups were analyzed using one‐way ANOVA, and the post hoc analysis using Tukey’s multiple comparison test. *p < 0.05, **p < 0.01, ***p < 0.001. [file KJM2-41-e70076-s002.jpg]
