# Supplementary material for: Long Noncoding RNA TOB1‐AS1 Represses Cervical Cancer Cell Proliferation, Invasion, and Migration via the MicroRNA‐27a‐3p/Thioredoxin‐Interacting Protein Molecular Axis
Source: Kaohsiung J Med Sci. 2025 Jul 16;41(11):e70076. doi: 10.1002/kjm2.70076 (PMC12622466; doi:10.1002/kjm2.70076)
Supplement: Supplementary file 2 — Data S1. Supporting Information. [file KJM2-41-e70076-s001.docx]

**Results**

To investigate whether miR-27a-3p had a regulatory effect on TOB1-AS1, HeLa cells were co-transfected with oe-TOB1-AS1 and miR-27a-3p mimics, while CaSki cells were co-transfected with si-TOB1-AS1 and miR-27a-3p inhibitor. The RT-qPCR analysis revealed that compared to the oe-AS1 + mimics-NC group, TOB1-AS1 expression was declined in the oe-AS1 + miR mimics group, and it was increased in the si-AS1 + inhibitor group group compared to the si-AS1 + inhibitor-NC group (all *P* < 0.05). These results demonstrated that miR-27a-3p exerted a regulatory effect on TOB1-AS1.
